# Mechanism of cellular rejection in transplantation

**DOI:** 10.1007/s00467-008-1020-x

**Published:** 2010-01-01

**Authors:** Elizabeth Ingulli

**Affiliations:** 1 Department of Pediatrics, University of California, San Diego, CA USA; 2 University of California, San Diego, 9500 Gilman Drive MC 0815, La Jolla, CA 92093 USA

**Keywords:** Allograft, Children, Rejection, T lymphocytes, Transplantation

## Abstract

The explosion of new discoveries in the field of immunology has provided new insights into mechanisms that promote an immune response directed against a transplanted organ. Central to the allograft response are T lymphocytes. This review summarizes the current literature on allorecognition, costimulation, memory T cells, T cell migration, and their role in both acute and chronic graft destruction. An in depth understanding of the cellular mechanisms that result in both acute and chronic allograft rejection will provide new strategies and targeted therapeutics capable of inducing long-lasting, allograft-specific tolerance.

**Learning objectives**:

1. To review recent advances in understanding the mechanisms of allograft rejection
2. To outline the current data on allorecognition and its role in allograft rejection
3. To discuss current therapeutics targeting costimulatory pathways
4. To briefly discuss recent data on the role T regulatory and memory T cells play in alloimmune responses

## Introduction

Transplantation of solid organs has emerged as a viable therapeutic modality for the treatment of a variety of ailments, such as end stage renal disease. Acute allograft rejection is understood as an impediment to long-term allograft survival, increasing the risk of developing chronic rejection and decreasing allograft half-life by 34% [1]. With the widespread use of potent immunosuppressive drugs, early graft loss due to acute rejection has decreased dramatically; however, current immunosuppressive protocols have not reduced the rates of graft loss due to chronic rejection and have increased the risk of serious complications, such as life-threatening infections and cancers [2].

Rejection of solid organ allografts is the result of a complex series of interactions involving coordination between both the innate and adaptive immune system with T cells central to this process. The ability of recipient T cells to recognize donor-derived antigens, called allorecognition, initiates allograft rejection. Once recipient T cells become activated, they undergo clonal expansion, differentiate into effector cells, and migrate into the graft where they promote tissue destruction. In addition, CD4 T cells help B cells produce alloantibodies. Here, we will review the components of an anti-allograft adaptive immune response.

## Allorecognition

Antigens that activate the immune system against the allograft, i.e. alloantigens, are both major and minor histocompatibility antigens. The major histocompatibility complex (MHC), located on chromosome 6 in humans, encodes the human leukocyte antigens (HLA), which are polymorphic molecules responsible for eliciting the strongest of responses to allogeneic tissues. The genes in this region encode for class I (HLA-A, -B, -C) and class II (HLA-DR, -DP, -DQ) molecules. The function of MHC molecules is to present foreign antigens to T cells. It has been known for more than 30 years that the T cell receptor (TCR) present on the surface of the T cell interacts with a peptide bound in the groove of the MHC molecule present on the surface of the antigen presenting cell. CD8 T cells recognize peptide/MHC class I complexes. MHC class I molecules are constitutively expressed on the surface of virtually all nucleated cells. CD4 T cells recognize peptide/MHC class II complexes. MHC class II molecules are constitutively expressed on the surface of professional antigen presenting cells, but expression can be induced on many cell types with activation.

Minor histocompatibility antigens are proteins that are expressed in some individuals in the population but not others, thereby creating potential antigenic differences between donors and recipients. This occurs, for example, when proteins encoded on the Y chromosome (H-Y) from male grafts induce an anti-Y response in females [3]. In theory, a polymorphism of any protein between donor and recipient, as is the case for certain enzymes and surface receptors that can be processed and presented on self-MHC, can potentially elicit an anti-graft response. Any non-MHC gene that encodes epitopes capable of binding to both MHC class I and class II molecules and inducing both CD4 and CD8 T cell responses can be considered a minor histocompatibility gene. CD8 T cells [4, 5] and, more recently, CD4 T cells [6] specific for minor antigens have been isolated from humans and rodents and have been shown to play an important role in the rejection of solid organs and corneal transplants as well as causing graft-versus-host disease after bone marrow transplantation [3, 7].

**Fig. 1:**
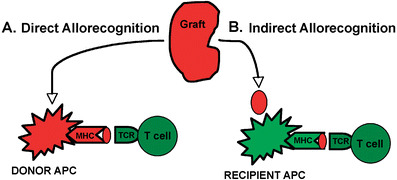
Two distinct pathways of allorecognition. **a** Direct pathway of allorecognition. Dendritic cells migrate from the graft to secondary lymphoid tissues to activate T cells. **b** Indirect pathway of allorecognition. Graft proteins are processed by recipient dendritic cells and presented to T cells. *APC* Antigen-presenting cell, *TCR* T cell receptor, *MHC* major histocompatibility complex

Unique to transplant immunobiology is the idea that alloantigen recognition can occur via two distinct pathways, both of which focus on the source of the antigen presenting cells (donor versus recipient). The direct pathway of allorecognition describes the ability of T cells to “directly” recognize intact non-self MHC molecules present on the surface of donor cells (Fig. 1a). The indirect pathway of allorecognition describes the ability of T cells to recognize donor MHC molecules that are processed and presented as peptides by self-MHC molecules (Fig. 1b). The recognition of intact donor MHC molecule(s) elicits a potent anti-graft immune response while processed MHC peptides and minor histocompatibility antigens elicit a slower tempo, less intense immune response.

## Direct pathway of allorecognition

T cells will respond vigorously when mixed in culture with MHC-disparate stimulator cells, i.e. the mixed lymphocyte reaction [8]. This in vitro response is thought to reflect the propensity for acute rejection [9], and the ability to detect an alloresponse with the mixed lymphocyte culture is generally believed to be due to the high precursor frequency of alloreactive T cells within the periphery. Mature, naïve T cells in circulation survive a selection process in the thymus that ensures that their TCR has a low but significant affinity for a self-peptide/MHC molecule but a high affinity for foreign peptides associated with self-MHC molecules [10, 11]. Recent studies have shown that the weak interaction between self-MHC and the TCR is required for the survival of naive T cells in the periphery [12]. The inherent affinity of the TCR on mature T cells for self peptide-MHC complexes probably explains the high frequency of T cells within any individual that cross react with high affinity to a closely related allo-MHC molecule [13, 14].

Direct recognition does not conform to the classic rules of self-MHC restriction. Mounting evidence suggests that the structural similarity between certain MHC molecules is ‘close enough’ to allow T cell receptor ligation and to trigger TCR signaling and subsequent activation [13, 15]. Alloreactive T cells are thought to recognize polymorphic residues on allogeneic MHC regardless of the peptide bound to it [16–18]. However, evidence also exists to support the notion that peptide binding facilitates a diverse T cell response [19, 20]. It is possible that, in the setting where the donor MHC is structurally very different from the recipient MHC, recognition may occur regardless of the peptide bound. Alternatively, if the donor MHC is structurally similar to the recipient MHC, recognition may occur through the peptide/MHC complex [21–23].

In order for recipient T cells to directly respond to intact allo-MHC molecules, cells from within the graft must migrate out of the graft [24] to make direct contact with recipient T cells within secondary lymphoid tissue. The first evidence that graft-derived cells participate in the alloimmune response was reported by Lafferty and colleagues [25] and was termed the passenger leukocyte theory. In their studies, the culture of thyroid cells prior to transplant prolonged graft survival, an effect that was thought to be due to the removal of the donor-derived passenger leukocytes from the graft [25–28]. Follow-up studies in rat renal allografts confirmed prolonged survival upon removal of passenger leukocytes but provided evidence that acute rejection could be induced with the injection of donor dendritic cells (DC) [29, 30].

Dendritic cells are professional antigen-presenting cells (APC) [31] that have been implicated as the passenger leukocyte responsible for inducing an acute anti-allograft response [32]. In their immature state, DC are abundant within peripheral tissues and organs where they are ideally positioned to capture antigens. Upon receiving inflammatory signals, such as interleukin (IL)-1β [33], tumor necrosis factor (TNF)-α [34], and CD40 [35–37], these cells undergo a maturation process and migrate via afferent lymphatics to the paracortex of lymph nodes [38, 39] where naïve and central memory T cells primarily reside [40, 41]. Dendritic cells, unlike macrophages and B cells, are potent stimulators of naïve T cells due in part to their high levels of class I and class II MHC and costimulatory molecules. Once activated, graft-specific T cells infiltrate the graft where they are capable of recognizing the alloantigens directly on the graft parenchyma. Over time, however, donor APC are depleted from the graft, and the response is predominated by recipient DC that migrate into the graft and continuously pick up antigens from the graft and present processed peptides to T cells through the indirect pathway [42, 43].

## Indirect pathway of allorecognition

In contrast to the direct pathway, the indirect pathway of allorecognition describes recipient APC presenting foreign MHC molecules in the form of peptides associated with self-MHC molecules [29]. This is the means by which most exogenous antigens enter the immune system and are recognized by T cells. Therefore, all proteins in donor grafts that differ from the recipient are potential antigens capable of inducing an anti-graft response. Three mechanisms of antigen delivery can be postulated to occur via this allorecognition pathway. First, antigens from the graft can be shed into the circulation and engulfed by recipient DC that reside within secondary lymphoid tissue. Second, donor cells can migrate to secondary lymphoid tissue where they are engulfed by recipient DC. Third, recipient APC can migrate into the graft, pick up antigens, and then migrate to secondary lymphoid tissue.

Evidence to support the indirect pathway as a viable means by which rejection is initiated comes from studies in which peptides derived from donor MHC molecules have been eluted from the binding grooves of recipient MHC molecules [44]. Furthermore, in vitro detection of an indirect response has been found to correlate with clinical rejection episodes in solid organ recipients [45, 46]. In addition, Auchincloss and colleagues [42] showed that in a situation where APC from a skin graft were incapable of activating CD4 T cells, rejection involving recipient CD4 T cells still occurred. In these studies, it was presumed that recipient MHC class II+ cells activated the recipient CD4 T cells. Immature DC have the unique ability to produce not only peptide/MHC class II complexes from exogenous antigens, but also peptide/MHC class I complexes [44, 47–49]. It is therefore conceivable that both kinds of peptide/MHC complexes derived from donor antigens could be presented to both CD4 and CD8 recipient T cells via this pathway.

There are two distinct differences between the direct and indirect pathway that merit clarification: first, the precursor frequency for T cells activated through the indirect pathway is significantly lower; second, the effector arm of the immune response within the graft differs from the direct pathway. When the donor and recipient differ, for example, at the MHC class I level, cytotoxic CD8 T cells specific for donor peptides bound to recipient MHC class I molecules (indirect pathway) would be unable to kill parenchymal cells of the graft because the graft cells express donor and not recipient MHC class I molecules. Therefore, recipient APC would have to migrate into the graft and take up residence, or the graft would have to share MHC identity with the recipient [50]. T cells specific for donor peptides bound to recipient MHC molecules could damage the graft indirectly by producing cytokines that through a bystander effect would damage graft cells [51].

In the setting of MHC-identical transplantation, the expression of the same MHC molecules by the donor and recipient blurs the distinction between direct and indirect donor antigen presentation. The stimulus for rejection in this situation is donor minor antigen peptide MHC class I and class II complexes. These complexes can theoretically be produced by donor cells themselves or by recipient phagocytes after engulfing donor cells or debris. The magnitude of the T cell response would be small at first because the frequency of T cell clones reactive to processed peptides from donor MHC proteins presented by recipient DC is orders of magnitude lower than the frequency of T cells specific for allogeneic MHC molecules [52]. However, a recent study demonstrated that the frequency of graft-specific T cells activated via the indirect pathway influences the ability of the costimulatory blockade to be effective in promoting graft survival [53].

## Innate alloimmunity

Every renal allograft undergoes a degree of ischemic reperfusion injury during transplantation and, as a result of this injury, the innate immune system is activated. Activation of the innate immune response can initiate acute rejection and contribute to the development of chronic allograft nephropathy. The mechanism by which ischemia reperfusion injury promotes rejection is likely to be multifactorial. Studies have shown that reperfusion injury activates both a cellular response and humoral factors of the innate immune system.

Central to the ischemia injury are reactive oxygen species (ROS) [54]. Reactive oxygen species are directly toxic to cells inducing apoptosis and/or necrosis. The greater the ischemic insult, the more ROS generated and, consequently, the greater the toxic effect to the graft. The ROS trigger activation of caspases, such as caspase 3, resulting in apoptosis [55]. In addition, ROS induce activation of chaperoning proteins, which are ligands to toll-like receptors (TLRs). These proteins can be secreted from stressed or damaged cells (i.e. heat shock protein 72 and high-mobility group box 1), or they can be altered matrix proteins (i.e. hyaluronan fragments) [56, 57]. By binding to TLR4 or TLR2, these ligands activate immature TLR-expressing DCs and/or vascular endothelium [58–60]. Toll-like receptor-mediated DC activation induces DC to migrate from grafts to secondary lymphoid tissues to initiate an adaptive alloimmune response [61].

Oxidative injury also facilitates signaling through adaptor molecules. Adaptive molecules, such as MyD88 and TRIF, have been shown to play a role in the development of acute rejection [62, 63]. Signaling through these adaptor molecules has been reported to promote chemokine expression within grafts, such as IP-10. IP-10 is a central chemokine that promotes T cell recruitment into allografts [64]. Studies using MyD88- and/or TRIF-deficient allografts demonstrate impaired donor-derived DC migration and less graft cell damage [65]. Redundancy within the innate immune response exists. Dendritic cells can also be activated upon reperfusion by activated natural killer (NK) cells, NK T cells, and Tγδ cells. Blocking any of these signaling pathways during reperfusion of a transplanted allograft could blunt activation of the adaptive immune response and prevent graft rejection.

## Costimulation

T cell activation is central to graft rejection. Tissue destruction occurs due to direct T cell-mediated lysis of graft cells, T cell activation of accessory cells, alloantibody production, and/or complement activation. Some studies have implicated CD4 T cells as sufficient on their own to result in complete graft destruction [66], while other studies have suggested that CD8 T cell activation alone results in acute rejection [67]. It is now understood that T cells require at least two signals to become optimally activated and develop effector function [68–70]. Alloantigen-specific signals are delivered through the T cell receptor (Signal 1), and antigen-nonspecific signals are delivered through accessory or costimulatory molecules (Signal 2). Although not graft specific, these costimulatory signals are essential for the development of potent anti-graft responses. Blocking costimulatory pathways at the time of T cell activation with the intention of prolonging graft survival and inducing tolerance has been an area of intense research over the past two decades. Lack of costimulation at the time of antigen presentation has been shown to induce T cell deletion, unresponsiveness (anergy), suppression, regulation, and/or immune deviation.

One of the most intensely studied costimulatory pathways involved in allo-T cell activation is the CD28/B7 pathway. CD28 is expressed on resting T cells, and its ligands B7.1 (CD80) and B7.2 (CD86) are expressed on APC. Signaling through CD28 lowers the threshold of TCR signaling to promote T cell proliferation, cytokine production, and differentiation. Several groups have shown in animal models that blocking CD28 signaling on T cells prevents both acute [71–73] and chronic [74] allograft rejection and can induce anergy [75, 76]. Cytotoxic T lymphocyte-associated antigen 4 (CTLA4), a homolog to CD28, is up-regulated on activated T cells and binds to CD80 and CD86 with greater affinity than CD28 [77]. This molecule is antagonistic to CD28 and transmits an inhibitory signal turning off T cell activation [78, 79]. However, blocking the CD28 pathway alone has been less effective in promoting tolerance in certain situations [80, 81]. This may be explained by recent data suggesting that Signal 1 and Signal 2 can be sufficient to stimulate CD8 T cell proliferation and clonal expansion, but that a third signal delivered early in the response is essential for naïve CD8 T cells to develop optimal effector function [82, 83]—especially if the T cells are activated through the indirect pathway [84].

For the rejection of grafts mismatched for minor histocompatibility antigens, cooperation between CD4 and CD8 T cells is thought to be required for maximal graft rejection. CD4 T cells have been shown to facilitate CD8 T cell differentiation by direct cell-to-cell contact or by producing effector cytokines, such as IL-2 and IFN-γ, that directly support CD8 T cell differentiation and killing [85]. Alternatively, it is possible that CD4 T cells act indirectly through a dendritic cell to be a more potent stimulator [35–37] or suppressor of CD8 T cell responses [86]. This indirect effect could be mediated through CD154 expression on CD4 T cells and CD40 expression on dendritic cells [36, 84].

The CD154/CD40 costimulatory pathway has been widely studied in animal models of transplantation. CD154 is expressed on activated T cells, while CD40 is constitutively expressed on APC. CD154/CD40 interaction was initially shown to be important for humoral immune responses, but it has also been shown to enhance T cell responses [87]. Blocking CD154 alone has been shown to inhibit both acute and chronic rejection in animal models [88–94], but when used in combination with CD28/B7 blockade, the effect on prolonging graft survival can be synergistic [90, 95].

## Costimulatory blockade

Building upon the knowledge learned from animal studies, researchers have been able to develop novel therapeutics currently in clinical trials for transplantation. Costimulatory blockade offers selective but long-lasting, graft-specific immunosuppression without nephrotoxicity and the possibility of inducing tolerance. The first pathway targeted was the CD28 pathway. In an attempt to block CD28 signaling, a soluble fusion protein was developed that consists of the extracellular binding domain of CTLA4 fused with the Fc domain of human immunoglobulin (Ig)G1, creating abatacept (CTLA4Ig) [96]. Abatacept binds to both CD80 and CD86, blocking CD28 engagement and T cell activation [73]. However, although transplantation studies in rodents demonstrated efficacy, studies in nonhuman primates did not live up to the expectation of inducing tolerance [97]. The failure of abatacept was thought to be secondary to a fast off-rate from CD86; consequently, a second generation agent, LEA29Y or belatacept, was created by codon-based mutagenesis, and it did demonstrate superior binding to CD80 and CD86 than abatacept [98]. In nonhuman primate renal transplant studies, belatacept was better at preventing acute rejection episodes than abatacept [99]. Belatacept is currently in phase III human clinical trials to determine if blocking this pathway in humans can promote graft survival and allow reduced exposure to calcinurin inhibitors [100]. Preliminary data suggests it may also prevent the development of chronic rejection [101]. Other agents, such as agonists to CD28, TGN1412, have been developed to target this pathway with the intention of expanding a regulatory T cell population. These studies have been abandoned at present due to the resultant cytokine storm and shock-like symptoms [102].

Targeting other costimulatory pathways, such as the CD154/CD40 pathway, are very appealing because of the potent ability to block T cell activation as well as antibody production that has been demonstrated in small animal models. Initial studies in nonhuman primates demonstrated long-term kidney allograft survival using anti-CD154 [93]. However, anti-CD154 (hu5C8) treatment in humans and nonhuman primates resulted in thromboembolic complications not observed rodent studies. This has been attributed to the expression of CD154 on human but not mouse platelets [103]. Current areas of intense investigation are focused on alternative costimulatory and inhibitory molecules that would target T cell adhesion and T cell memory [104, 105].

## Regulatory T cells

Regulatory T cells are considered to be essential mediators of peripheral tolerance by maintaining immune homeostasis, preventing autoimmunity, and regulating inflammation. Studies have shown a positive correlation between regulatory T cell function and allograft survival [106–108]. T regulatory cells suppress immune responses by a number of mechanisms: production of suppressor cytokines, direct suppression of effector cells, and modulation of DC maturation and function. Harnessing the power of T regulatory cells is appealing as a potential tolerizing strategy in transplant recipients; however, markers that consistently identify and isolate regulatory T cells in vivo have been elusive.

Although both CD4 and CD8 T cells have been shown to demonstrate suppressive function, much attention has focused on a subpopulation of CD4 T cells that express high levels of CD25, the a subunit of the IL-2 receptor. CD4+CD25+ T regulatory cells have been identified in peripheral blood samples of tolerant liver allograft recipients [109] and within tolerated allografts [110]. Adoptive transfer of CD4+CD25+ T regulatory cells has been shown to prevent graft rejection and graft-versus-host-disease (GVHD) in animal models [111]. There are difficulties, however, in using CD25 as a marker for regulation. For example, because CD25 is up-regulated on activated T cells, its sustained expression on regulatory T cells could be confused with recently activated T cells. A transcription factor known as forkhead box P3 (FoxP3) was recently identified in regulatory T cells. This transcription factor is required for the development, maintenance, and function of T regulatory cells [112, 113]. It has proven to be a consistent marker to identify T regulatory cells in murine models but has not been as consistent in humans. In humans, transient expression of FoxP3 has been observed during T cell activation [114], and FoxP3 has recently been identified in inflamed and rejecting allografts [115, 116]. The expression of FoxP3 on graft infiltrating cells has also been associated with donor-specific hyporesponsiveness and less chronic changes on biopsy [117]. This conundrum emphasizes the continued need for further characterization of T regulatory cells to identify an exclusive marker of regulatory cells in humans. Despite this difficulty, CD4+CD25+FoxP3+ T cells with suppressive function can be generated de novo with costimulatory blockade, such as CTLA4Ig, anti-CD154, and non-depleting anti-CD4 [118–120], and in vitro with rapamycin [121, 122]. It has been recently shown that donor-derived T regulatory cells can inhibit CD4 T cells responses as well as recipient-derived T regulatory cells [123]. These data entertain the possibility of cell therapy using regulatory T cells generated in vitro.

## Memory T cells

Memory T cells can be divided into central memory and effector memory subsets based on their circulation pattern and functional responsiveness. Memory T cells have been shown to be more sensitive to antigen, function more rapidly, produce effector cytokines, survive longer, and show less dependence on CD28 costimulation than their naïve counterparts [124–129].

Memory T cells specific for alloantigens can be generated after exposure to blood transfusions, pregnancy, rejection of a previous transplant, homeostatic proliferation, and heterologous immunity. Homeostatic proliferation refers to the division of peripheral T cells in a lymphopenic environment in the absence of antigenic stimulus. This occurs after a situation where T cells are depleted, i.e. after viral infection or immunotherapies, and has been shown to be dependent upon recognition of self antigens [130] and the presence of factors such as IL-7 [131]. After undergoing homeostatic proliferation, naïve T cells will change their phenotype to that of a memory cell and display some of the functional properties of memory cells [132–134]. Heterologous immunity refers to the generation of memory cells to infectious antigens that cross react with alloantigens [135, 136]. This would result in allo-specific memory in the absence of specific exposure. In transplant studies, it is clearly understood that memory T cells, however they are generated, pose a significant barrier to inducing tolerance to allografts [129, 137–140]. As humans age, the proportion of memory phenotype T cells increases. Thus, a better understanding of how to target this cell population and the designing novel of therapies that inhibit these cells would be beneficial.

## T cell migration

Naïve T cells and central memory cells circulate between blood and secondary lymphoid tissue and are excluded from non-lymphoid tissues, such as the skin, gut, and lung. This migration pattern is guided mainly by the cell surface expression of specific homing molecules, such as selectins, integrins, and chemokine receptors. Activation of naïve lymphocytes occurs within secondary lymphoid tissue [40, 87, 141, 142]. Upon activation and differentiation, marked changes in the homing behavior of lymphocytes are observed as a direct result of changes in the cell surface expression of homing molecules. The interactions between these molecules and their ligands or receptors triggers a sequential and coordinated series of events; leukocyte rolling, stopping, and transmigration enable T cells to move from the blood across endothelial cells into peripheral tissues.

The new combination of cell surface molecules expressed on differentiated T cells enables access to tissues that were previously ‘off limits’. For example, activated T cells lose the expression of CD62L and CCR7, which prevents cells from re-entering peripheral lymph nodes. At the same time, they express increased levels of VLA-4 and LFA-1, which facilitates binding to endothelial cells at sites of inflammation. Different sites of inflammation express different adhesion molecules to select for different cell populations. This is evident from studies in which blocking CD62E and CD62P inhibited T cell infiltration into the skin [143–149]. In addition, much work has been done to characterize the chemokines expressed in the rejection of heart allografts [150]. They have been divided into early events, related to the ischemia and reperfusion injury of grafts, and late events, which are related to the immune response [151]. The specific chemokines found to be important for lymphocyte trafficking in rejecting heart grafts are CXCL9 (MIG), CXCL10 (IP-10), and CXCL11 (I-TAC) [150]. Neutralizing chemokines or blocking their receptors has been shown to prolong graft survival and prevent graft infiltration in animal models [152, 153].

The change in homing phenotypes appears to be determined during the transition from a naïve to memory T cell [154]. In fact, recent data suggest that it occurs during the initial activation and differentiation in secondary lymphoid tissue and that the unique microenvironment of the secondary lymphoid tissue draining various tissue sites directs the homing phenotype imprinted on T cells activated at that site [154–156]. Further studies have linked this education to the resident DC within lymphoid tissue [156]. Thus, the difference in T cell responses between vascularized and nonvascularized grafts could be explained, in part, by differences in the migratory capacity and thus the ability of cells to infiltrate grafts.

## Chronic rejection

Chronic rejection is now the leading cause of graft failure in pediatric renal transplant recipients. Organs undergoing chronic rejection display many of the features of healing wounds, including fibroblast, endothelial cell, or epithelial cell proliferation and collagen deposition within the graft parenchyma and blood vessels; all of these processes result in interstitial fibrosis, ischemia, and the loss of graft function [157, 158]. Although risk factors can be identified, the pathophysiology of chronic rejection remains poorly understood. Both immunologic and nonimmunologic injuries have been shown to play a role in the development of chronic rejection.

Major histocompatibility complex-mismatched grafts, which undergo acute rejection in the absence of immunosuppression, undergo chronic rejection in rodents even if acute rejection is prevented [159, 160]. Allografts that are depleted of passenger leukocytes survive acute rejection only to succumb to chronic rejection [42]. It is postulated that chronic rejection occurs after donor DC are replaced by recipient DC within the allograft. Thus, chronic rejection of vascularized organs is thought to occur via the indirect pathway [45, 46]. As mentioned above, this type of T cell stimulation cannot damage parenchymal cells of graft origin directly because these cells express donor MHC molecules. Recipient T cells responding to allopeptide/self-MHC complexes on recipient APC that enter the graft can only cause bystander damage by producing cytokines or other soluble mediators. Several studies implicate the Th2 cytokines (IL-4, IL-5, IL-6, IL-10, and IL-13) as having a role in chronic rejection or fibrosis [161, 162]. Injection of Th2 cells induces chronic allograft rejection in immunodeficient recipients [163]. Dermal fibrosis in skin grafts undergoing chronic rejection has been shown to be blocked by treatment with anti-IL-4 antibodies [164]. Similarly, fibrosis induced by chemical injury is dependent on IL-4 [165]. These effects could be explained by the findings that IL-4 stimulates the production of extracellular matrix proteins by fibroblasts and that IL-4 and IL-10 inhibit macrophage production of metalloproteinases that digest extracellular matrix proteins [166, 167]. Type 2 cytokine-producing T cells would also be expected to promote antibody production by B cells [168]. Results from several studies indicate that chronic rejection-related fibrosis is dependent on anti-graft antibodies, which may cross-link surface antigens on endothelial cells and thereby cause the production of growth factors and complement activation [169, 170]. Although these studies suggest that Th2 cytokines enhance fibrosis, another study found that chronic heart graft rejection was inhibited in Stat4-deficient mice that are impaired with respect to the generation of Th1 cells [171]. Given the therapeutic implications, it is essential to resolve the nature of the relationships between graft antigen-reactive T cells, their products, and fibroblast proliferation and collagen production. One aspect of chronic rejection that is currently under intense investigation is the role of alloantibodies in the development and progression of chronic rejection.

## B cells and alloantibodies

B cells and anti-HLA antibodies have recently been shown to play an important role in both acute and chronic allograft rejection. The presence of CD20+ B cells and plasma cells infiltrating allografts has been found to correlate with irreversible acute rejection episodes [172, 173]. The ability to detect circulating anti-HLA antibodies in patients and the association of tissue deposition of C4d, a complement split product, has revitalized the study of alloantibodies. Acute antibody-mediated rejection was added to the Banff criteria in 2003 [174] and has been recently updated [175]. The diagnosis of acute (or chronic) antibody-mediated rejection depends upon the presence of three criteria: (1) serologic evidence for circulating anti-donor antibodies, (2) C4d+ staining in peritubular capillaries, and (3) morphological evidence of acute (or chronic) tissue injury. Intense (>1+), widespread (>50%) C4d staining of peritubular capillaries is both a sensitive and specific marker of acute antibody-mediated rejection. In rodent models, C4d deposition within kidney allografts is alloantibody dependent and begins as a focal process progressing to diffuse peritubular capillary staining. In nonhuman primate studies, circulating donor-specific alloantibodies and deposition of C4d in peritubular capillaries lead, in most cases, to chronic transplant glomerulopathy and arteriopathy [176].

Rituximab, a chimeric anti-CD20 monoclonal antibody therapy, has been shown to be effective in some but not all cases of acute humoral rejection [177, 178]. This may be due in part to the elimination of CD20-expressing B cells but not plasma cells, which do not express CD20 [179]. In chronic inflammatory situations, ectopic lymphoid structures can form within grafts and are not responsive to rituximab therapy even though circulating alloantibodies are reduced [180].

B cells not only damage grafts by producing anti-graft antibodies, but they have recently been shown to infiltrate grafts and present graft-derived antigens to alloreactive T cells via the indirect pathway of allorecognition [181]. Stimulation of B cells by antigen in the presence of T cells helps drive naïve B cell proliferation and differentiation into memory B cells and plasma cells [182]. Memory B cells survive in lymphoid tissue in the absence of antigen and, upon challenge with antigen, they respond rapidly with robust proliferation and antibody production. Plasma cells, in contrast, home to the spleen and bone marrow, are terminally differentiated, and are thought to be responsible for the circulating levels of antibodies. It is debated whether human plasma cells last a lifetime or, alternatively, need to be replenished from the B cell pool [183]. In contrast to plasma cells, memory B cells require reactivation to produce antibodies. Thus, the lack of circulating alloantibodies may not reflect lack of sensitization but rather may reflect the lack of active antibody-producing plasma cells. New therapies aimed at targeting alloantigen-specific memory B cell activation and alloantibody production would be advantageous.

## Immunological monitoring

**Fig. 2:**
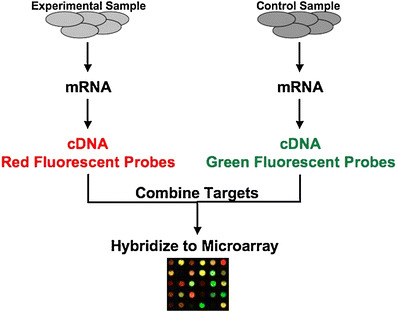
DNA microarray analysis DNA from experimental samples is purified and fluorescently labeled and then hybridized to immobilized probes on the array to determine alterations in gene expression

Analysis of gene expression using genomic microarrays can complement clinical research and may provide insights into disease pathogenesis. A microarray is a high-throughput technology that consists of a series of small nucleic acid sequences or oligonucleotides (called probes) that are attached to a solid surface (i.e. glass slide or a silicon chip). Millions of probes can be contained in one array. DNA and RNA of experimental and/or control samples can be extracted, labeled, and hybridized to the probes under high-stringency conditions (Fig. 2). Probe–target hybridization is usually detected and quantified by fluorescence-based detection methods to determine relative abundance of DNA sequences in the target (ratio of test to reference sample). DNA microarrays can be used to measure changes in the levels of gene expression or to detect single nucleotide polymorphisms. The technology is reproducible across multiple samples and when large numbers of genes are analyzed, it can be used to identify patterns of gene expression within disease states that are believed to correlate with functional changes at the protein level. This technology can produce an overwhelming amount of data that must be analyzed using sophisticated data analysis software. The current cost and complexity of this technology precludes its use as a screening tool in the clinical setting.

DNA microarrays have been applied to peripheral blood and renal allograft biopsy samples of pediatric patients with and without graft dysfunction in the posttransplant period [184]. Several gene expression patterns are altered during acute rejection episodes, chronic allograft nephropathy, and infection. Three molecular categories have been identified during acute rejection episodes that appear to correlate with graft function and survival. For example, biopsy samples that demonstrate enhanced B cell-related genes appear to have the worst outcomes. Since this initial study, many studies have subsequently been performed in both humans and mice investigating acute rejection, chronic allograft nephropathy, operational tolerance, minimum immunosuppression, infections, and drug toxicity [185]. In some cases, these have resulted in the identification of molecular subtypes that can predict outcome and response to treatment; in other cases, potential novel therapeutic targets have been identified. The expectation from these studies is to move from a ‘one-size fits all’ to a more personalized approach to posttransplant immunosuppressive regimens.

## Summary

The benefits learned at the bench and, in particular, in small animal models are beginning to translate to the bedside. Novel therapeutics currently in clinical trials in humans have originated from basic studies investigating the requirements of T cell activation. It is clear that costimulatory blockade alone, while highly effective at blocking activation of naïve T cells, may be effective in blocking memory T cell responses, and current T cell depletion-based therapies may, in fact, promote memory T cell development. Recent studies have suggested that memory T cells pose the next barrier to overcome in the quest to induce allograft-specific tolerance. Once again, we turn to the bench.

## Questions

### (Answers appear following question list)

1. Which of the following statements is true?
  - A six-antigen matched kidney is at risk for chronic rejection.
  - Naïve T cells are activated directly in the allografts where they mediate acute rejection.
  - Memory T cells have increased susceptibility to costimulatory blockade.
  - Plasma cells are depleted with rituximab therapy.
2. Which statement is true of the indirect pathway of allorecognition?
  - Activation of T cells occurs by recognition of intact donor MHC molecules.
  - The pathway thought to be involved in chronic rejection.
  - Passenger leukocytes migrate from the graft to activate T cells in lymphoid tissue.
  - The frequency of T cells specific for a given alloantigen activated via this pathway is high.
3. Costimulation
  - is the synergy between CD4 and CD8 T cells that results in acute rejection.
  - is the signaling that results in optimal T cell activation.
  - can easily be blocked in humans to result in tolerance.
  - refers to induction therapy.
4. Memory T cells directed against an allograft can be formed after
  - pregnancy
  - a viral infection
  - depletional anti-T cell antibodies
  - all of the above
  - none of the above

#### Answers

1. a
2. b
3. b
4. d
